# Epigenome and Epitranscriptome: Potential Resources for Crop Improvement

**DOI:** 10.3390/ijms222312912

**Published:** 2021-11-29

**Authors:** Quancan Hou, Xiangyuan Wan

**Affiliations:** 1 Zhongzhi International Institute of Agricultural Biosciences, Shunde Graduate School, Research Center of Biology and Agriculture, University of Science and Technology Beijing (USTB), Beijing 100024, China; 2 Beijing Engineering Laboratory of Main Crop Bio-Tech Breeding, Beijing International Science and Technology Cooperation Base of Bio-Tech Breeding, Beijing Solidwill Sci-Tech Co., Ltd., Beijing 100192, China

**Keywords:** epigenetics, epitranscriptomics, epigenome editing, epitranscriptome engineering, crop improvement

## Abstract

Crop breeding faces the challenge of increasing food demand, especially under climatic changes. Conventional breeding has relied on genetic diversity by combining alleles to obtain desired traits. In recent years, research on epigenetics and epitranscriptomics has shown that epigenetic and epitranscriptomic diversity provides additional sources for crop breeding and harnessing epigenetic and epitranscriptomic regulation through biotechnologies has great potential for crop improvement. Here, we review epigenome and epitranscriptome variations during plant development and in response to environmental stress as well as the available sources for epiallele formation. We also discuss the possible strategies for applying epialleles and epitranscriptome engineering in crop breeding.

## 1. Introduction

Since the birth of agriculture, human beings have never stopped domesticating plants. For thousands of years, we have selectively bred crops with desirable traits, such as high yield, nutritious, biotic- and abiotic resistance, etc. Most modern crop varieties, including rice (*Oryza sativa*), wheat (*Triticum aestivum*), and maize (*Zea mays*), are obtained from conventional breeding approaches, which rely on the selection and collection of favorable alleles from the offspring of crossed varieties. Although modern varieties provide nutritious crops with high yields, the global human population is predicted to reach 10 billion by 2050 and will exceed our ability to meet the nutritional needs of humans around the world [1].

Breeders and plant scientists have been applying different strategies to accelerate the breeding process. For example, by extending photoperiods and controlling temperatures, the so-called “speed breeding”, the generation times of wheat, barley (*Hordeum vulgare*), chickpea (*Cicer arietinum*), pea (*Pisum sativum*), and canola (*Brassica napus*) have been significantly shortened [2,3]. The explosion in available reference pangenomes allow breeders to use marker-assisted selection and genome selection easier, facilitating efficient phenotyping and genotyping plant materials [4]. The automated and machine-learning-assisted high-throughput phenotyping systems enable the efficient screening, selection, and evaluation of large populations [5,6].

Crop genetic engineering by adding or editing genetic information can increase yield and improve crops in adverse environments. For instance, overexpression of *OsDREB* genes leads to enhanced drought tolerance in rice [7]. Higher expression of *OsIPA1* by overexpression or mutation at the miR156 and miR529 target sites has improved grain yield and immunity in rice [8]. Increased OsGRF4 abundance elevates grain yields of rice and wheat grown in moderate nitrogen-supply [9]. Genetic variation generated through genome editing such as CRISPR/Cas can be indistinguishable from naturally occurring variation and thus should be readily accessible for commercialization. Using the CRISPR-Cas9 system, multiple endogenous genes that function in plant architecture, plant immunity, nitrogen use, and other pathways have been manipulated to improve crops directly. Many genes have been targeted by using genome editing platforms to engineer disease resistance [10]. Double knockout of Microrchidia *MORC1* and *MORC6a* using CRISPR/Cas9 significantly increases the resistance of barley to biotrophic (*Blumeria graminis*) and necrotrophic (*Fusarium graminearum*) plant pathogenic fungi [11]. Knockout of FAD2 genes by CRISPR/Cas9 leads to increases in oleic acid and total monounsaturated fatty acid composition with concurrent decreases in undesirable polyunsaturated linoleic and linolenic fatty acid content [12]. CRISPR/Cas9-mediated gene editing of *GmJAGGED1* increased yield in a low-latitude soybean variety [13]. With the help of genome editing, a remarkable work to domesticate wild allotetraploid rice de novo into a new staple cereal has been reported recently. Six agronomically important traits were rapidly improved by editing *O. alta* homologs of the genes controlling these traits in diploid rice [14]. Such strategies described above will greatly accelerate the breeding process and strengthen world food security.

Thus far, plant breeding has made use of genetic variation, but epigenetic factors, such as DNA methylation, can also be heritable and can contribute to breeding. Epigenetics is the study of heritable changes in genome function that are not attributed to alterations of the DNA sequence but involve the control of DNA packaging to switch genes on or off. In plants, many biological processes are associated with epigenetic regulation, such as vernalization, paramutation, transgenic silencing, imprinting, etc. Epitranscriptomics has revealed that RNA modifications are critical posttranscriptional regulators of gene expression affecting that cell differentiation and development [15]. Knowledge on epigenetic and epitranscriptomic control for plant development and biotic and abiotic resistance is accumulating, and epigenetic and epitranscriptomic editing for crop breeding is emerging [16,17,18,19]. In this review, we summarize recent progress on understanding the contribution of epigenomic and epitranscriptomic variations to plant traits and discuss the potential applications for crop breeding.

## 2. Epigenetics and Epitranscriptomics

**Figure 1:**
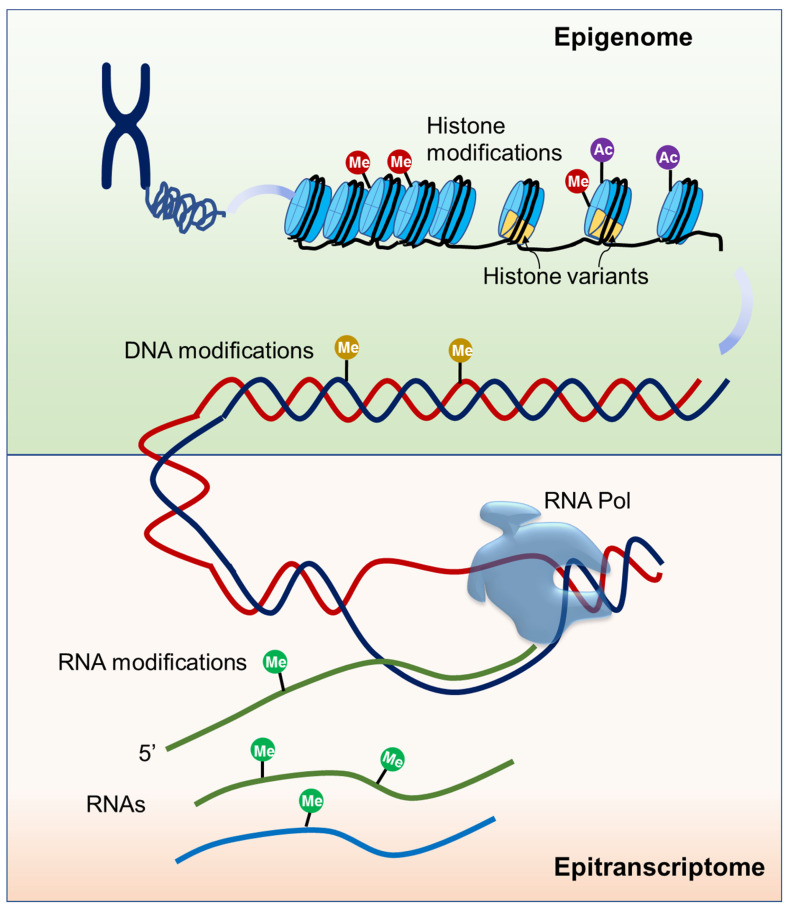
Schematic of epigenome and epitranscriptome. Epigenome is mainly composed of modifications of DNA and histone proteins. Epitranscriptome is composed of all biochemical RNA modifications.

Epigenetic mechanisms play essential roles in all kingdoms of life and these mechanisms generally include DNA and histone modifications, histone variants, and some non-coding RNAs (ncRNAs) [20] (Figure 1). It is known that each of the four DNA bases could be chemically modified and at least 17 DNA modifications have been discovered, among which 5-methylcytosine (5mC) is the best characterized [21]. In plants, *de novo* DNA methylation is established by the RNA-directed DNA methylation (RdDM) pathway and DNA methylation on the sequence contexts CG, CHG (where H = A, C or T), and CHH is maintained by different DNA methyltransferases [22]. 5mC can be actively removed by 5-methylcytosine DNA demethylases, a kind of DNA glycosylase/lyase family enzymes [22]. 5mC is dynamically regulated and tightly associated with other chromatin elements, exerting widespread effects on gene expression during plant development and in response to environmental factors. The effects highly depend on the location of the methylation relative to the gene. 5mC present over the transcription start site often leads to gene silencing while the gene body 5mC has minimal effects on gene expression [23,24]. Recently, N6-methyladenine (6mA) modification has also been identified as a new epigenetic mark in plants [25,26,27]. Unlike the silencing function of 5mC in gene promoters, the distribution of 6mA on genomes is divergent among species and its effect needs to be investigated. Though information on 6mA is less known, the available evidence suggests that it functions in plant development, tissue differentiation, and gene expression regulation [26,27].

Modifications at histone residues mainly include methylation, acetylation, phosphorylation, and ubiquitination. These covalent modifications on histones called the “histone code” can alter chromatin structure or recruit interaction effectors, influencing transcriptional activity. The different types of histone modifications play different roles in specifying chromatin function. For example, histone H3 with tri-methylation on lysine 4 (H3K4me3) and 36 (H3K36me3) is often distributed on actively expressed genes and associated with euchromatin, whereas H3K27me1 and H3K9me2 are usually present within heterochromatic regions [28]. In addition to histone methylation, other histone marks such as acetylation, phosphorylation, and ubiquitination are also associated with gene expression regulation. Acetylation can occur at many lysine residues of H2A, H2B, H3, and H4 [29]. Histone acetylation relaxes the chromatin structure and leads to transcriptional activation, while histone deacetylation condenses the chromatin structure, often resulting in transcriptional repression [30]. Histone marks are established, recognized, and removed by specific proteins or protein complexes that are referred to as the writers, readers, and erasers, respectively [28,31]. For example, H3K4me3 deposition is catalyzed by the methyltransferases Arabidopsis Trithoras-like Protein1 (ATX1) and ATX2 in Arabidopsis [32]. H3K27me3 is catalyzed by the polycomb repressive complex 2 (PRC2) via the histone methyltransferases Curly Leaf (CLF), Swinger (SWN), and Medea (MEA), and can be recognized by the PRC1 complex through the reader proteins Like Heterochromatin Protein 1 (LHP1), Early Bolting in Short Day (EBS), and Short Life (SHL) [33,34]. The removal of histone lysine methylation is catalyzed by jumonji C (JmjC) domain-containing proteins and lysine-specific demethylase1 (LSD1)-like proteins [28].

In addition to DNA and histone modifications, three main types of RNA are also subjected to biochemical modifications (Figure 1). Although it was revealed long ago that chemical modifications are critical for ncRNAs to facilitate their full function, modifications on mRNA are recently disclosed to be important for RNA metabolism [35]. So far, about 160 chemical modifications have been discovered in RNA, and N6-methyladenosine (m6A) is one of the most abundant modifications on mRNA. m6A amount is estimated to account for 0.1–0.4% of the total adenosine in cellular mRNA, approximately 2–3 sites per transcript [36]. m6A mRNA modification is catalyzed by a conserved multicomponent methyltransferase complex in eukaryotes. In Arabidopsis, mRNA adenosine methyltransferase MTA, MTB, Fkbp12 Interacting Protein 37KD (FIP37), Kiaa1229/Virlizer (VIR), and Hakai have been reported to have adenosine methyltransferase activity, and knockout or knockdown any of these factors result in decreased m6A levels [37,38,39,40]. ALKBH family proteins were identified as m6A demethylases to remove methyl groups [41,42]. YTH domain-containing proteins were identified as reader proteins that bind to m6A-modified mRNA in vivo and affect mRNA stability in Arabidopsis [43]. Another mRNA modification, 5-methylcytosine (m5C), has been detected in different eukaryotes, including Arabidopsis [44]. The distribution of m5C on mRNA is still unclear. RNA bisulfite sequencing (RNA-BisSeq) analysis revealed that m5C is abundant in 3’UTRs while m5C-RIP-seq analysis showed that m5C is enriched in coding sequence [44,45]. RNA (C5-cytosine) methyltransferase (RCMT) family proteins have been identified as m5C mRNA methyltransferases in Arabidopsis. So far, there has been no m5C-binding protein identified in plants [46]. Recently, the N4-acetylcytidine modification (ac4C) has been identified as a new reversible RNA modification present in tRNA, rRNA, and mRNA, and plays a vital role in mRNA stability and translation fidelity [47]. In humans, ac4C is catalyzed by the N-acetyltransferase 10, and SIRT7 has been identified as a deacetylase [48]. However, nothing is known about ac4C mRNA modification in plants. Several other RNA modifications such as N6,2′-O-dimethyladenosine (m6Am), 8-oxo-7,8-dihydroguanosine (8-oxoG), and pseudouridine (Ψ) have also been shown to influence the mRNA stability and consequently affect translation efficiency [49].

## 3. Epigenomic and Epitranscriptomic Changes during Development

Much evidence has indicated that epigenetic and epitranscriptomic modification profiles vary in plant-specific organs and cell types. DNA methylation in the CHH context displays significant differences among leaves, flowers, and ovules, in line with small RNA abundance at corresponding sites [50]. The DNA methylation variations could be partially attributed to the tissue-specific expression of Classy (CLSY) genes, which encode chromatin remodelers that are involved in the RNA-directed DNA methylation (RdDM) pathway by facilitating RNA polymerase IV (Pol IV) recruitment and small RNA generation [50]. A comparison of DNA 5mC methylomes of the shoot apical meristem revealed that CHG methylation and CHH methylation were increased after the transition from vegetative to reproductive growth in Arabidopsis and rice, respectively [51,52]. Although most root cell types have similar 5mC landscapes, columella displayed genome-wide hypermethylation in the CHH context [53]. The increased mCHH in columella is mainly distributed in transposable elements, and this might be a mechanism to keep the neighboring stem cells silenced during the root development [54]. Several studies identified 5mC changes in the male reproductive cells [55,56,57]. Some regions gain methylation in the sex cells in the CHH context via RdDM, and genes within these regions are upregulated in an RdDM mutant in meiocytes but not in leaves. This suggests RdDM is required for the silencing of these genes, specifically in the male sex lineage [57]. Transposable elements (TEs) have reduced 5mC DNA methylation in the vegetative nucleus (VN) but not in sperm cells (SC), resulting in the generation of 21 nucleotide siRNAs from *Athila* retrotransposons in VN. The VN-generated siRNAs could further target TEs in gametes and ensure gamete TEs are silenced, which is essential for the silencing of TE in the next generation [55]. Similarly, the meiocytes’ nurse cells generate TE-derived small RNAs that can distribute into meiocytes and lead to TE silencing by RdDM [56]. DNA methylation alteration in gametes could be significant for the inheritance of DNA methylation and may provide potential targets for generating DNA methylation variation in crop species. In addition, DNA methylome alterations have been documented in soybean development and during tomato fruit ripening [58,59]. The level of 6mA also shows a dynamic pattern in plant development. In Arabidopsis, 6mA accumulation during vegetative development is significantly correlated with the upregulation of gene expression [26]. In rice, the mutation of Deficient in DNA Methylation 1 (DDM1) that significantly decreased the level of 6mA resulted in downregulation of gene expression [27]. These results suggest 6mA is associated with actively expressed genes, which is in contrast to 5mC. However, 6mA is also enriched on transposable elements and over the pericentromeric heterochromatin regions [26,27].

Some histone marks also show dynamic changes in plant development. For example, the profile of H3K27me3 varies among different tissues of maize [60], and genes of Arabidopsis are differentially marked by H3K27me3 during cell type transitions [61]. Knockout of components of the polycomb group (PcG) chromatin remodeling complex responsible for catalyzation of H3K27me3 results in abnormal development [62], suggesting H3K27me3 plays an essential role in defining plant cell fate. H3K27me3, H3K4me3, and gene expression profiling in Arabidopsis in different root cells and guard cells demonstrated that H3K27me3 dynamics regulate cell identity [61,63]. A comparison between young and mature leaves revealed a relationship between gene expression changes and H3.3 content on the affected genes [64].

Measurement of the level of mRNA modifications by using different approaches revealed that mRNA modifications display dynamic patterns in plant development. In Arabidopsis, transcriptome-wide m6A-seq revealed that 33.5% of transcripts showed differential m6A methylation between leaves, flowers, and roots [65]. Thin-layer chromatography analysis showed m6A levels differ among different tissues, with a high ratio (1.5%) in young seedlings and relatively lower ratios in leaf (0.9%) and root (0.6%) [37]. Analysis by liquid chromatography coupled with tandem mass spectrometry (LC-MS/MS) revealed m5C levels ranged from 0.01% in rosette leaves to 0.036% in siliques, with m5C abundance slightly increasing from 3-day-old (0.027%) to 15-day-old (0.033%) seedlings [44]. RNA bisulfite sequencing of siliques, shoots, and roots tissues of Arabidopsis showed that most m5C sites were tissue specific, and only 15 sites were commonly methylated between all three tissue types [45]. In rice, 1792 and 6508 tissue-specific m6A-modified genes were identified in callus and leaves, respectively [66]. Dynamic changes of mRNA m6A modification have also been observed during tomato fruit ripening [67]. Consistently, the transcript levels of writer, eraser, and reader coding genes vary in different tissues and during plant development [37,38,44]. Disruption of the components of writers, erasers, or readers would alter mRNA decay rates and often cause severe developmental problems. For example, the knockout of the genes encoding core m6A writer components results in embryonic lethality [37,68]. Loss of function of the m6A reader ECT2 affects mRNA stability degradation of the trichome development-related transcripts and leads to more extensively branched trichomes [43]. Mutations in *TRM4B*, encoding an m5C methyltransferase, display primary and lateral root development defects and decreased m5C levels on root development-related genes [44].

## 4. Epigenomic and Epitranscriptomic Changes in Response to Abiotic and Biotic Stresses

In the past decade, examining epigenomic changes upon various abiotic and biotic stress treatments has become a hot topic. Studies have revealed that epigenetic mark dynamics are associated with abiotic and biotic stress responses. Drought stress treatment globally changed the 5mC DNA methylation levels of the *P. trichocarpa* genome and altered the expression profiles of many drought-stress-responsive genes [69]. In rice, drought-induced genome-wide 5mC DNA methylation changes accounted for ~12.1% of the total site-specific methylation differences and 29% of the drought-induced DNA demethylation/methylation changes remain even after recovery [70]. On the contrary, other studies showed that the DNA methylome is stable in response to drought and excess light stress, in which a few 5mC DNA methylation changes were detected upon the stress treatment [71,72]. Studies also have shown that drought stress-induced gene expression is related to the alteration of histone modification dynamics. A recent study showed that the PRC1 complex negatively regulates drought resistance through H3K27me3 deposition on transcription factors of ANAC019 and ANAC055 and causes transcriptional repression of the TFs and their target genes such as Vegtative Storage Protein 1 (VSP1) [73].

High-salinity treatments enrich the active histone marks H3K9K14ac and H3K4m3 but decrease repressive marks H3K9m3 and H3K27me3 on salt stress-responsive genes [74,75]. A rapid increase in H3 Ser-10 phosphorylation, a histone mark related to chromatin density, was also observed in Arabidopsis leaves subjected to high salinity [76]. High-affinity K^+^ Channel 1(HKT1) controls Na^+^ entry and high-affinity K^+^ uptake and is associated with plant salt tolerance [77]. Expression of the Arabidopsis HKT1 is activated by salt treatment. A decrease in the repressive mark H3K27me3 on the gene body of *HTK1* may be the cause of the salt induction [78]. In wheat, the expression of *TaHKT2;1* and *TaHKT2;3* was downregulated under NaCl stress in shoot and root tissues. The downregulation was correlated with the increase in cytosine methylation on the coding regions of *TaHKT2;1* and *TaHKT2;3* [79]. Similarly, the expression of another salt stress-induced transcription factor *MYB74* was regulated epigenetically. Under normal conditions, heavy cytosine methylation was observed in a region around the transcription initiation site of *MYB74* and this region is targeted by 24-nt siRNAs. However, methylation of this region was decreased to an undetectable degree when plants were exposed to salt stress, and the expression of *MYB74* was upregulated consequently [80].

Several studies have revealed that cold and heat stress also have impacts on epigenetic marks. Heat stress can decrease DNA methylation and increase chromatin accessibility at some transposons and DNA repeats [81]. The Arabidopsis Suppressor of DRM1 DRM2 CMT (SDC) that regulates the expression of a number of long-term heat-stress-responsive genes is epigenetically silenced by the RdDM pathway under normal conditions but is activated by heat stress [82]. Heat stress induces the deposition of H3K4me3 and H3K9Ac on several heat shock proteins encoding genes, *HSP18*, *HSP22.0*, and *HSP70*, which play a crucial role in conferring heat tolerance. Kwon et al. found that cold stress leads to a decrease in H3K27me3 deposition in some cold-responsive genes, including *C repeat binding factor-cold responsive* (*COR15A*) and *Galactinol synthase 3* (*GOLS3*) [83]. Another study found cold treatment induces histone acetylation in the promoter regions of some *COR* genes, accompanying the expression activation of these genes [84]. Long-term cold treatment in rubber trees (*Hevea brasiliensis*) induced DNA demethylation on promoters of cold-related genes *HbICE1* and *HbCBF2* and elevated their transcriptional activities [85].

Epigenetic variations in response to biotic stress have also been reported. DNA methylation and histone modification dynamics have been monitored upon plant exposure to pathogens and changes in plant–pathogen interactions of some DNA methylation and histone modification factor mutants [86,87,88]. Pathogen-induced DNA demethylation occurs in promoters, gene bodies, and nearby TEs of defense-related genes, and the DNA demethylation is generally correlated with transcriptional activation of these genes [88]. For instance, Arabidopsis mutants (*met1* and *ddc*), which cause global cytosine methylation depletion, are more resistant to the bacterial pathogen *Pseudomonas syringae* pv. tomato DC3000 (*Pst*), suggesting that active demethylation is required for maintaining the genome methylome state in plant pathogen defense and DNA demethylation might be required for expression activation of defense genes. *Pst* treatment induces changes in cytosine methylation throughout the genome, and an analysis of all DMR (differentially methylated regions)-associated genes revealed that these genes are associated with plant defense and their demethylation is correlated with increased gene expression [86]. In addition to the DNA methylation dynamic changes, histone modifications are also involved in plant defense. Wheat histone deacetylase TaHDA6 interacts with the WD40-repeat protein TaHOS15, which promotes histone deacetylation of defense-related genes and suppresses wheat defense responses to the fungal pathogen *Blumeria graminis* f. sp. *tritici* (*Bgt*) [89]. The cytoplasmic effector PsAvh23 secreted by the soybean pathogen *Phytophthora sojae* PsAvh23 suppresses H3K9 acetylation on defense genes mediated by disrupting the assembly of the histone acetyltransferase (HAT) complex and increases plant susceptibility [90]. Infection by bacterial blight pathogen *Xanthomonas oryzae* pv. *oryzae* (*Xoo*) causes global H3 methylation on multiple lysine sites in the plant genome and induces JmjC domain-containing protein-encoding genes, which function as histone lysine demethylases. JmjCs further reduce H3K4me2/3 at promoters of the rice defense negative regulator genes such as *NRR*, *Os-11N3*, and *OsWRKY62*, thereby potentiating the rice defense response against *Xoo* infection [91].

Reports on epitranscriptomic dynamic changes in plant response to abiotic and biotic stress are emerging. Expression analysis revealed that m6A and m5C writer encoding genes are nearly constantly expressed upon different abiotic stress, indicating m6A and m5C methylation play a fundamental role in plant stress responses [92]. However, it was found that m6A is dynamically deposited on transcripts encoding proteins required for salt and osmotic stress responses, reducing RNA secondary structure, and thus stabilizing the transcripts and eventually increasing the protein levels [93,94]. m6A reader proteins ECT1 and ECT2 are found to be involved in the signaling transduction of various external stimuli by interacting with CIPK1 (Calcineurin B-Like-Interacting Protein Kinase1) [95]. ECT2 controls the cytosol mRNA fate by recognition of the m6A motif and allows it to relocate mRNA to stress granules upon heat exposure [96]. In Arabidopsis, the lack of the m6A eraser protein ALKBH9B decreases m6A removal from the alfalfa mosaic virus (AMV) genome and impairs viral accumulation and systemic invasion [42].

## 5. Sources for Epiallele Formation

The epiallele refers to a genetic locus with specific DNA or histone modifications that can arise from either genetic source or non-genetic factors [97]. Naturally occurring epialleles that are associated with agriculturally important phenotypes, including organ genesis [98], fruit ripening [99], and environmental adaptation [100], have been identified from different plant species. The exact origin of these epialleles is still unclear, and current knowledge suggests they are primarily generated from spontaneous epimutations through gains or losses of DNA methylation or histone modification stochastically [101]. The Linaria cycloidea-like gene (*Lcyc*) epimutation is the first example of a natural epiallele discovered in *Linaria vulgaris*, in which the fundamental symmetry of the flower is changed from bilateral to radial. There was no sequence change in the *Lcyc* epiallele, but the *Lcyc* locus is extensively methylated and transcriptionally silent in the mutant. The DNA methylation pattern is heritable and co-segregates with the mutant phenotype. However, the mutant phenotype occasionally reverts to the wild type during somatic development, correlating with the demethylation of *Lcyc*, which indicates epimutations can occur naturally and cause significant phenotypic changes in plants [102]. However, the exact causal factor of the *Lcyc* has not been investigated, probably due to the dysfunction of the normal demethylation pathway. Large-scale epigenetic changes can also result from genetic alterations such as through crossing or transposable element mobilization [103,104]. The Wassilewskija (WS) ecotype of Arabidopsis has four *phosphoribosylanthranilate isomerase (PAI*) genes, two of which are located together and form an inverted repeat. All four *PAI* genes of WS are methylated, whereas the Columbia (Col) ecotype has three singlet *PAI* genes with no methylation. All three Col *PAI* genes in the crossed offspring of WS and Col-0 gain methylation, and the methylation is stable over multiple generations even when the inverted repeat has segregated away. Such natural epialleles that convert the wild-type (paramutable) allele to a paramutagenic allele are also known as paramutations. It is assumed that paramutagenic alleles could generate small RNAs and convert other alleles to a repressed state through the RdDM pathway and the converted allele becomes paramutagenic itself when it encounters a paramutable allele [105].

Like genetic mutations, spontaneous somatic epimutations are common and the epimutation rate at CG dinucleotides is much higher than the genetic mutation rate [101]. The region-level epimutation rate is not linked to genetic mutations but depends on the chromosomal location, where chromosome arms and the centromere display the highest and lowest epimutation rates, respectively [106]. Genetic changes could lead to large-scale epigenetic changes and the resulting epimutation can be maintained even after the genetic change is lost [103]. Epigenetic changes could also result from the mistargeting of epigenetic modifiers, such as the acquisition of gene-body DNA methylation [107]. However, gene-body methylation usually does not have a functional influence on plants [24]. Transposable elements strongly influenced non-CG DNA methylation acquisition on its flanking sequence, indicating genetic variations determine natural DNA methylation variation [108]. A comparison of epimutation rates between *Populus trichocarpa* and Arabidopsis showed that the rates of epimutations per year in *P. trichocarpa* were lower than in Arabidopsis. However, the epimutation distribution patterns on genomic regions of the two species are similar. The lower epimutation rate of *P. trichocarpa* could be attributed to the few meristematic cell divisions during the tree lifespan. However, epimutations were accumulated year by year, suggesting the epimutations were accumulated from mitosis [109].

Chemical treatment can also trigger global epigenetic changes. For example, DNA demethylating compounds 5-AzaC (5-Azacytidine) and Zebularine can be incorporated into DNA and inhibit DNA methylation by trapping the DNA methyltransferases and mediating their degradation [110]. Many biological processes such as embryogenesis, shoot regeneration, and flowering require the expression activation of specific genes. Treatment with these demethylating compounds changes the hypermethylated gene promoters to the hypomethylated status and activates gene expression. 5-AzaC treatment has been widely used in tissue culture because it can induce somatic embryogenesis [111]. The treatment of 5-AzaC promotes the initiation of flowering and causes a profound influence on flower bud morphogenesis in *Salix viminalis*, which is correlated with the decrease in DNA methylation [112]. Other studies also showed 5-AzaC treatment increases transposon activity, reactivates silenced transgenes, and diminishes stress-induced transgenerational memory [113,114,115]. Histone deacetylase inhibitors such as Trichostatin A have also been used as epigenetically active substances for inducing somatic embryogenesis [116]. Although these epigenetically active chemicals could change the status of DNA methylation or histone acetylation and result in plant phenotypic or response alterations, the disadvantage of the chemical treatment is that their influence is global and does not specifically modify the locus of interest.

The creation of epiRILs (epigenetic recombinant inbred lines) is a good way to obtain epigenetic variations and link epialleles to phenotypes. In Arabidopsis, epiRILs have been created by crossing the *met1* mutant with wild-type plants. Crossing the progeny for several generations could cause each epiRIL to become a homozygote but with different DNA methylation patterns between epiRILs [117]. These epiRILs constitute a valuable library of epialleles and display extensive phenotypic variations, including altered flowering time and improved disease resistance [117]. Identifying the artificial epialleles associated with the specific phenotypes will provide a novel epigenomic source for breeding, especially for crops low in genetic diversity. However, it is challenging to create such an epiallele library for other plants. Attempts in rice and maize have shown they are sensitive to severe genome-wide DNA methylation alterations and cause deleterious phenotypic effects [118,119]. Therefore, new methods for moderately perturbing the DNA methylomes need to be developed for crops [120].

Clonal plant propagation through tissue culture is used widely for maintaining ideal varieties. However, it is known that tissue culture experiences dedifferentiation and redifferentiation and could induce epigenetic variation [121,122]. In maize, many stress-responsive loci were differentially methylated in tissue-culture-generated plants, implying tissue culture may act as natural stress [122]. Therefore, tissue culture could also provide an epiallele library with identical genetic information. Mostly, tissue culture-induced epialleles are deleterious. Clonal propagation of high-performing hybrid oil palm via tissue culture can generate many clones with the same phenotype at the vegetative stage, but some clones displayed abnormal floral phenotypes and destroyed the oil productivity years later. An epigenome-wide association study revealed a DMR that is correlated with the deleterious trait. The loss of DNA methylation of a transposable element within the intron of the *EgDEF1* gene leads to aberrant transcripts of a floral identity gene [123]. Thus, understanding the epigenetic mechanism underlying the phenotype helps identify novel beneficial epialleles and avoid deleterious epialleles.

## 6. Epigenome and Epitranscriptome Engineering for Crop Improvement

**Figure 2:**
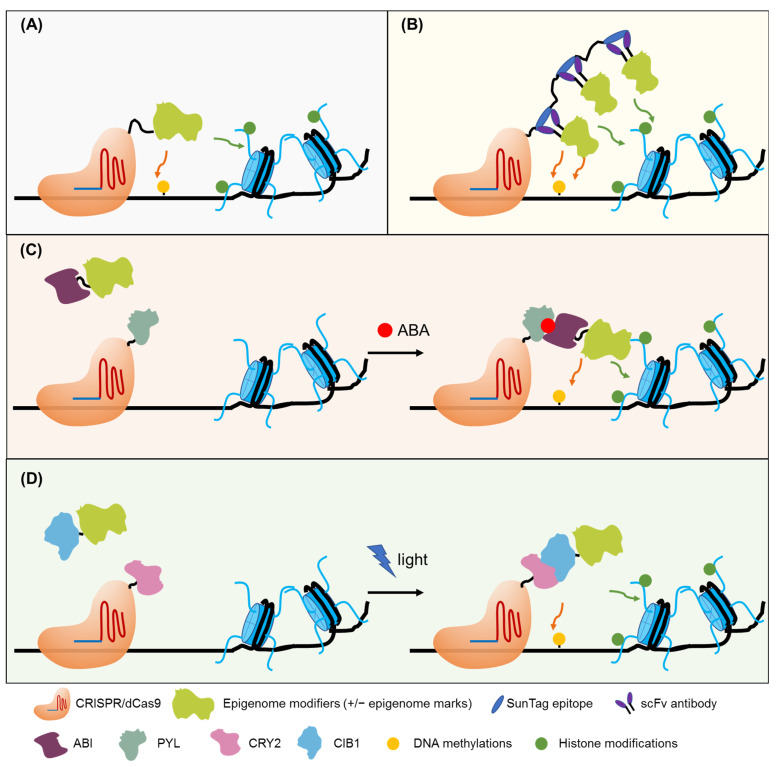
Epigenome editing tools. (**A**) Direct epigenome editing. Fusions of epigenome modifiers to deactivated Cas9 (dCas9) can be directed to specific loci and cause epigenetic changes of interest. (**B**) Enhanced epigenome editing. dCas9 is fused to SunTag epitopes and the single-chain variable fragment (scFv) is fused to epigenome modifiers. Multiple copies of scFv-epigenome modifiers can be directed to specific loci and cause epigenetic changes of interest. (**C**) Chemically inducible epigenome editing. ABA mediates the interaction of ABI and PYL to direct epigenome to dCas9-gRNA-targeting sites for epigenome editing. (**D**) Light-inducible epigenome editing. Light induces the interaction of CRY2 and CIB1 to direct epigenome modifiers to dCas9-gRNA-targeting sites for epigenome editing.

Recently, epigenome editing tools that specifically target a genome locus to change epigenetic modifications (cytosine de/methylation or histone tail de/methylation, de/acetylation, etc.) have been developed, enabling precise generation of artificial epialleles. These approaches were designed by fusing epigenetic modifiers or an interacting platform that can recruit the epimodifiers to nuclease-deficient genome editing tools, which guides the fused functional module to a predefined site and directly cause localized epigenome changes (Figure 2A,B). The zinc-finger (ZF) protein, transcription activator-like effector protein, and nuclease-dead CRISPR-associated protein 9 (dCas9) were commonly used for specific DNA sequence targeting. A chemically inducible dCas9 system has been successfully used in human cells [124] (Figure 2C), and a light-inducible dCas9 system was proposed to be adapted for epigenome editing [125] (Figure 2D). Successful applications of these epigenome editing tools have been shown at the *FWA* locus in Arabidopsis. The *Flowering Wageningen* (*FWA*) is a flowering repressor, and its promoter has tandem repeats that can be methylated or demethylated, resulting in gene silencing or activation, respectively [126]. The demethylated epiallele *fwa* displayed a delayed flowering phenotype. The ZF protein fused with RdDM components such as SUVH2, SHH1, NRPD1, RDR2, DMS3, or RDM and directed to the *FWA* promoter induces DNA methylation at the target sites [127,128]. Interestingly, co-targeting of ZF–DMS3 and ZF–NRPD1 enhanced the targeted methylation, suggesting multiple silencing factors have a synergistic effect when they are simultaneously recruited to a defined site [128]. Fusing a ZF or a dCas9 with the catalytic domain of the human DNA demethylase TET1 also led to efficient demethylation of the targeted *FWA* promoter [129]. Induced methylation or demethylation at the *FWA* promoter, which results in the creation of early or late phenotypes, are heritable traits, even when the epigenome editing module was segregated away, suggesting the stable creation of the epialleles [130]. Besides the *FWA* locus, when the fusion protein ZF-TET1 targeted the methylated regions of the *CACTA1* transposon, this also resulted in targeted demethylation and changes in expression [129].

**Figure 3:**
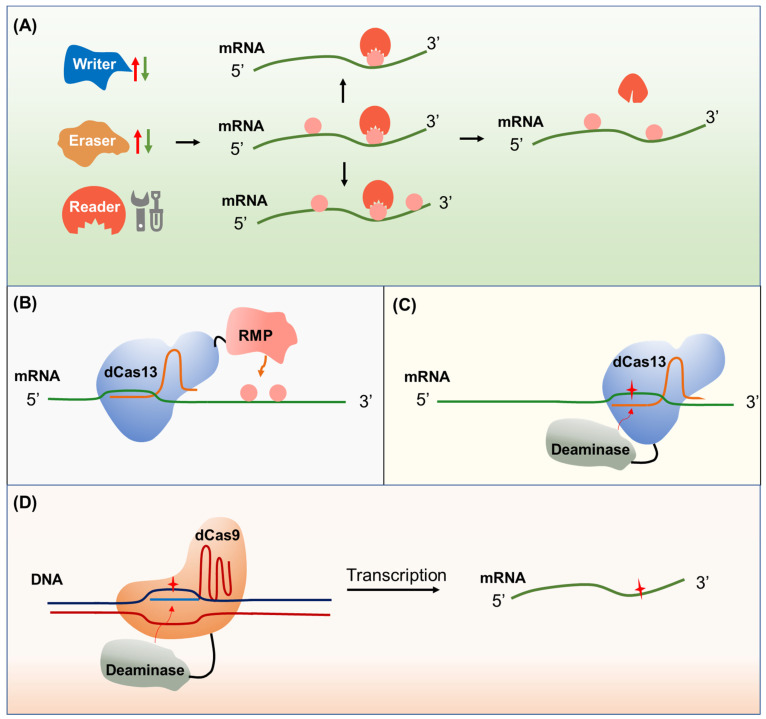
Epitranscriptome engineering tools. (**A**) Modulating the activity of RNA modification proteins by promoting or inhibiting the RNA modification writer or eraser proteins or manipulating the RNA modification reader proteins to trigger global RNA modification changes. (**B**) Direct epitranscriptome editing. Fusions of RNA modification proteins (RMP) to deactivated Cas13 (dCas13) can be directed to specific transcripts and cause epigenetic changes of interest. (**C**) Epitranscriptome editing through RNA base editing. Fusions of deaminase to deactivated dCas13 can be directed to the specific transcript for RNA base editing. The resulting synonymous mutations might cause RNA modification changes. (**D**) Epitranscriptome editing through DNA base editing. Fusions of deaminase to dCas9 can be directed to a specific locus for DNA base editing. The resulting synonymous mutations might further cause RNA modification changes.

Some RNA modifications have indispensable roles in plant development and tolerance to various environmental stresses, which are closely associated with agricultural traits [131]. For instance, m6A mRNA modification regulates strawberry fruit ripening [132]. OsNSUN2-mediated m5C mRNA modification has been shown to enhance rice adaptation to high-temperature stress [133]. m6A mRNA modification plays a vital role in salt-stress tolerance in Arabidopsis [134]. Thus, epitranscriptome manipulation has great potential for improving crop traits. Recent studies revealed that harnessing m6A regulation could remarkably improve economically important traits in crops [16,132,135]. Transgenic expression of a human RNA demethylase FTO (fat mass and obesity associated) in rice and potato stimulates root meristem cell proliferation, tiller bud formation, promotes photosynthetic efficiency, and results in ~50% increases in yield and biomass. Mechanistically, FTO causes substantial m6A demethylation of both mRNA and repeat RNA in the transgenic plants. m6A demethylation of plant repeat RNA further induces chromatin openness and subsequently causes a global transcriptional upregulation of tissue-specific genes encoding proteins that play functional roles in root cell proliferation, tiller formation, and photosynthetic efficiency [16]. Overexpression of *PtrMTA* encoding a component of the m6A methyltransferase complex that participated in the formation of m6A methylation exhibits enhanced poplar tolerance to drought stress. Poplar plants that overexpression of *PtrMTA* displayed an increased density of trichomes and a more developed root system than that of the wild type [135]. In strawberry, the overexpression of *FveMTA* or *FveMTB*, encoding m6A methyltransferases, accelerates fruit ripening, while the suppression of either delays fruit ripening, providing a good example of fruit maturity control through epitranscriptome manipulation [132]. Strategies for epitranscriptome engineering have been proposed from different angles (Figure 3): (1) Manipulating the activities of RNA modification-related proteins, including writers, readers, and erasers, by generating gain-of-function or loss-of-function mutants [136]; (2) specific RNA editing using fusions of catalytically inactivated dCas13 and RNA modification enzymes to create or remove RNA modifications on target sites [137]; and (3) eliminating specific RNA modification by manipulation at the DNA level, which requires precise base editors to generate synonymous mutation [138]. So far, only the first strategy has been applied in plants. The prerequisite for applying the other strategies needs a comprehensive understanding of the epitranscriptome at a single-base resolution and the associations with phenotypic outputs.

## 7. Conclusions and Perspectives

Both epigenome and epitranscriptome are plastic and vary during plant development and in response to environmental cues, and some agronomic traits have been known to be associated with epigenetic changes. Therefore, harnessing epigenetic and epitranscriptomic regulation may provide new additions to the crop breeding toolbox. However, the prerequisite for the application of epigenetic- and epitranscriptomic-based technologies in crop improvement is a deep understanding of epigenome and epitranscriptome regulation mechanisms. So far, our understanding of epigenetic and epitranscriptomic machinery in plants is mainly derived from model species. Delivering fundamental knowledge about epigenetic- and epitranscriptomic-mediated plant development and adaptability to environmental stress will significantly help to harness epigenetic variation for crop improvement.

**Figure 4:**
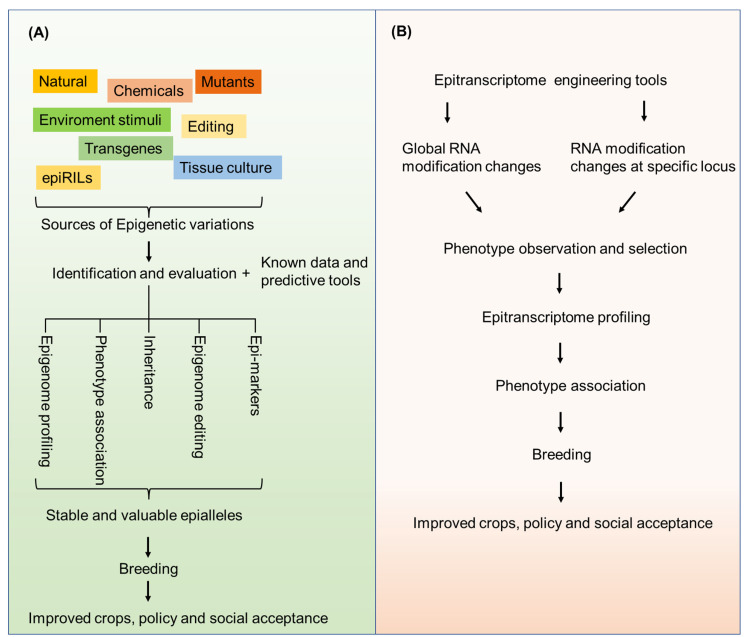
Routes for application of epialleles in crop breeding (**A**) and for application of epitranscriptome engineering in crop breeding (**B**).

Integrating epigenetics into crop improvement requires the induction of epigenetic variation, epiallele identification, and evaluation. Epigenome editing or epi-genomic selection may be required for epiallele creation and identification. The validated epialleles could then be introduced into elite cultivars through a breeding program (Figure 4). To discover and evaluate economic trait-associated epialleles is a major restriction for applying epigenetics to crop breeding. Identifying and establishing the relationships between epigenetic variations and associated plant phenotype changes is a challenge that requires excluding the effect from the underlying genetic variation. Furthermore, a detailed understanding of the stability and heritability of epigenetic variants is required for the stable improvement of agronomic traits. Nevertheless, the emerging technologies will greatly advance the process of application of epigenetics in crop breeding. Precise epigenome information obtained from the emerging single-cell profiling technology [139,140] and predictive tools based on deep learning [141] will allow for a better understanding of the dynamics of epigenome changes during plant development and response to the environment. The targeted epigenome editing tools allow efficient validation about whether specific epigenetic changes are causative for a phenotype.

To integrate epitranscriptomics into crop improvement, breeders must determine the influence of specific mRNA modification changes and then perform the epitranscriptome engineering using the strategies described above. Profiling modification sites at a single-base resolution, disclosing detailed involved components and regulatory mechanisms, and applying robust predictive tools and genome and epitranscriptome editing tools will help form a better understanding of the epitranscriptome and apply epitranscriptomics to crop breeding.
